# Real-time energy/mass transfer mapping for online 4D dose reconstruction

**DOI:** 10.1038/s41598-018-21966-x

**Published:** 2018-02-26

**Authors:** Peter Ziegenhein, Cornelis Ph. Kamerling, Martin F. Fast, Uwe Oelfke

**Affiliations:** Joint Department of Physics at The Institute of Cancer Research and The Royal Marsden NHS Foundation Trust, London, SM2 5NG UK

## Abstract

In this work we describe an ultra-fast, low-latency implementation of the energy/mass transfer (EMT) mapping method to accumulate dose on deforming geometries such as lung using the central processing unit (CPU). It enables the computation of the actually delivered dose for intensity-modulated radiation therapy on 4D image data in real-time at 25 Hz. In order to accumulate the delivered dose onto a reference phase a pre-calculated deformable vector field is used. The aim of this study is to present an online dose accumulation technique that can be carried out in less than 40 ms to accommodate the machine log update rate of our research linac. Three speed optimisation strategies for the CPU are discussed: single-core optimisation, parallelisation for multiple cores and vectorisation. The single-core implementation accumulates dose in about 1.1 s on a typical high resolution grid for a lung stereotactic body radiation therapy case. Adding parallelisation decreased the runtime to about 50 ms while adding vectorisation satisfied our real-time constraint by further reducing the dose accumulation time to 15 ms without compromising on resolution or accuracy. The presented method allows real-time dose accumulation on deforming patient geometries and has the potential to enable online dose evaluation and re-planning scenarios.

## Introduction

During the course of a radiation therapy treatment the patient anatomy may change from fraction to fraction or even within a single fraction. With the help of modern motion estimation techniques, dynamic multi-leaf collimator (MLC) tracking can be applied to adjust the treatment to the motion of the target volume. For lung treatments, a 4D CT planning image set is commonly used to derive a time-resolved representation of the patient motion for an average respiratory cycle. The 4D CT images are acquired and sorted into a number of phases (e.g. 10). These images of the temporally varying patient anatomy together with the machine logs from the linac lead to a motion-dependent dose distribution throughout the course of the treatment. This is especially important for MLC tracking deliveries, for which the interplay of target and machine motion is not known a-priori. The actually delivered dose can be reconstructed based on the temporal dose distributions and the motion model of the patient. Especially for hypo-fractionated lung treatments it is highly desirable to perform the dose reconstruction in real-time during irradiation as treatment margins are smaller compared to conventional treatments. In future, real-time dose reconstruction is expected to facilitate online adaptive radiotherapy (ART) techniques such as interventions in form of an online plan adaptation or interruption of the treatment in case certain quality indicators are violated.

In order to evaluate the actual cumulative dose distribution over the course of the treatment it is necessary to map the time-dependent dose distributions (referred to as moving phases or source phases) to a common reference geometry (referred to as target or reference phase). Motion during the treatment can also lead to deformation of anatomical structures in a patients body. The correct mapping of the dose at different times requires tracking of individual tissue elements from the moving phases to the reference phase. That information is provided by a displacement vector field (DVF) which correlates physical locations of the moving phase to locations in the reference phase. The DVF is typically generated by performing a deformable image registration (DIR). In a discretised voxel representation of the patient, the DVF then provides a displacement vector for each voxel from the moving phases to its respective location in the reference phase while the dose value of the source voxel and the target voxel are assigned to be equal.

Due to the deformable character of motion it is possible that two or more tissue elements from the source geometry are compressed to one element in the target geometry so that multiple voxels of one source phase have to be mapped to only one voxel in the reference phase. This scenario leaves an ambiguity of how multiple dose contributions to one reference voxel are handled. One solution to resolve this many-to-one mapping issue is to compute the average dose value of the source voxels and score it to the target voxel in the reference phase^1–3^. This method is often called direct dose mapping (DDM) and has been very popular due to its simplicity and low computational workload. An alternative approach which is motivated by the physical definition of dose determines the actual energy and mass which is transferred from the source to the reference phase and then divides energy by mass to obtain the deposited dose^4–9^. This method will be referred to as energy/mass transfer mapping (EMT). Various studies have shown that DDM and EMT can produce different results especially in heterogeneous tissue regions with a steep dose gradient: The DDM method has been compared^4^ with a direct voxel tracking method for Monte Carlo dose calculation which is defined to be the gold standard. The comparison showed a dose deviation between the two methods of up to 2% in a 4D phantom. A direct comparison between DDM and EMT has been carried out elsewhere^5^. The authors showed that DDM produced an average dose error of 1.1% along the beam while a maximal error of up to 24.9% in the penumbra of the beam was observed. Another study^6^ reported an average dose error of 7.3% for a lung IMRT plan when comparing DDM and energy/mass transfer mapping used in a Monte Carlo-based dose calculation scenario. A dose mapping strategy for clinical use has been implemented^9^ within the Eclipse treatment planning system (TPS). The authors compared the clinical manifestation of DDM and EMT for lung cancer treatment plans reporting up to 11.3% difference in the planning target volume (PTV) and 4.4% in internal target volume (ITV) minimum doses. For volumes with high dose and/or mass density gradients (i.e. situated 2 cm beyond the superior or inferior surface of the PTV) up to 7.7% difference in mean dose was observed between DDM and EMT.

Li *et al*.^9^ provides a detailed description of how to implement EMT for clinical use to account for inter- and intra-factional motion in the context of adaptive radiotherapy. However, the reference dose was accumulated offline in a post-processing step after the treatment. In this work we present an implementation of EMT which allows for direct online dose reconstruction on temporally varying anatomies (such as lung) based on the algorithm discussed by Li *et al*.^9^. We provide details of our implementation which allows to perform dose mapping on high resolution images with up to 25 Hz. We implemented the online EMT method within our in-house software environment^10^. We could demonstrated its usability for a real-time dose reconstruction workflow^11^ by applying the presented 4D dose accumulation algorithm to assess the performance of MLC tracking for clinical lung stereotactic body radiation therapy (SBRT).

## Materials and Methods

**Figure 1:**
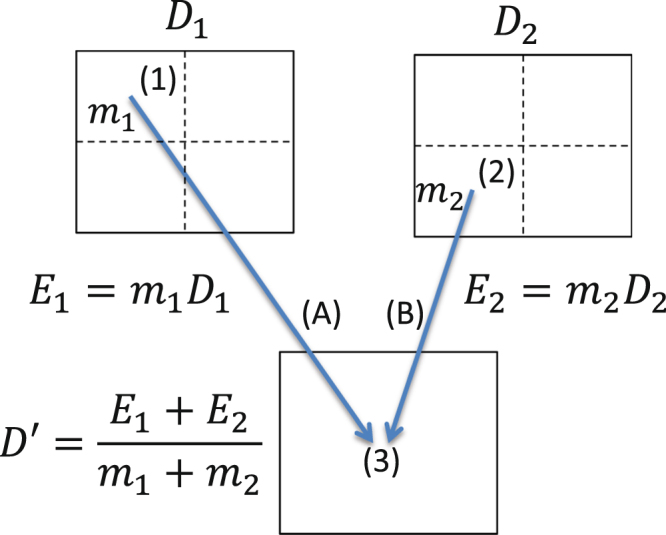
Energy/mass transfer mapping in deformable anatomy.

The energy/mass transfer mapping method is motivated by the physical definition of dose. It determines the energy and mass, which is transferred between a moving phase and the reference phase, separately. The accumulated dose in the reference grid is then obtained by dividing the energy and mass transferred to each voxel. This bypassed ambiguities which arises when dose values from multiple source voxels need to be accumulated to one voxel in the reference grid due to tissue deformation. An example of EMT mapping in deformable anatomy is given in Fig. 1.

The figure shows how two locations (1) and (2) from a moving phase are mapped to the same dose voxel (3) in the reference phase. Locations on the moving phase are defined by image voxels (dotted lines) which are stored at twice the resolution as the dose voxels (solid lines) in this example. Both grids are aligned rectilinearly so that 4 image voxels are comprised within one dose voxel on each slice of the moving CT. The mapping is described by two elements of the displacement vector field (A,B). The mass contained in a location can be retrieved from the CT data set. The energy transferred from both locations *E*_1_ and *E*_2_ is calculated from the mass of the image voxel and the dose of the overlapping moving phase voxel. The energy contributions are gathered in the target reference voxel and divided by the transferred mass to estimate the accumulated dose.

**Figure 2:**
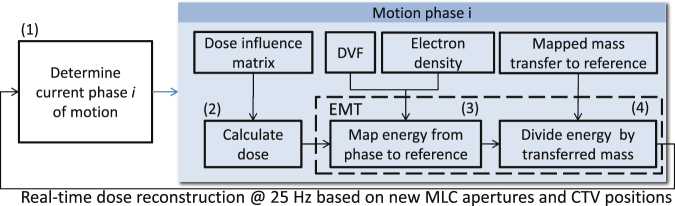
Real-time dose reconstruction workflow during tracked step and shoot IMRT delivery.

The general EMT mapping process is divided into three main steps. First, the energy and mass of every phase image voxel is calculated using the dose from the corresponding phase dose grid and the density contained in the image voxel. Second, energy and mass are accumulated in the reference grid according to the displacement vectors. Third, the accumulated dose is derived by dividing the transferred energy and mass for each voxel in the reference grid. The EMT-based dose accumulation is embedded into our research dose accumulation software environment^10^. Our in-house TPS, DynaPlan^11^, performs a dose reconstruction every 40 ms (25 Hz) based on the reporting frequency of actual delivered MLC apertures by our Elekta Agility research linac. A typical workflow of the dose reconstruction in lung using tracking is shown in Fig. 2.

Prior to each re-calculation step the respiratory phase *i* is determined according to the current target position (1). Based on the last reported MLC shapes the phase dose is calculated using a dose influence matrix data set (2). The resulting dose cube is then fed forward into the EMT module which first performs the energy mapping (3) using the DVF of phase *i* before dividing the energy by the transferred mass (4) to yield the expected accumulated dose. The dose influence matrix data set, the DVF and mass transfer information is pre-calculated for each phase of the patient movement. The dose calculation of the moving phase (2) is based on the algorithms introduced in^12^ and can be performed in about 10 ms^10^. The EMT runtime is dominated by (3) the energy mapping while (4) dividing energy by mass is a simple loop over the voxels of the reference dose cube.

Performance on modern computational hardware is achieved by exploiting parallelism on multiple levels. In this study we propose three CPU-based implementations for the energy mapping (Fig. 2 process (3)) which are characterised by their degree of parallelism. The next subsection describes an efficient but serial (non-parallel) EMT algorithm. It illustrates the general idea of implementing EMT on modern CPUs and serves as a baseline for the parallel implementation presented in following section and the vectorised implementation. The vectorised implementation includes the thread-level parallelism as well.

### Serial energy/mass transfer implementation

**Figure 3:**
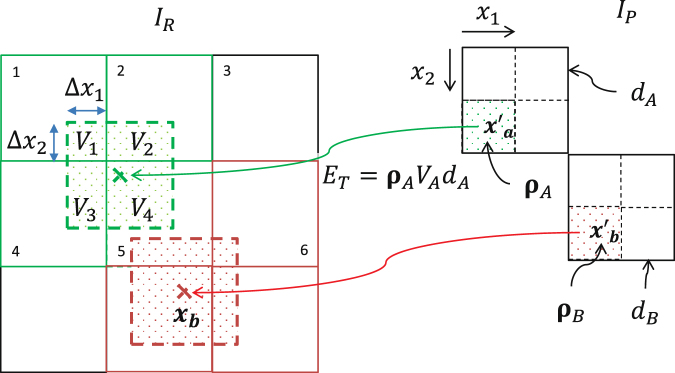
Energy/mass transfer process illustrated in detail by mapping two locations *x*_*a*′_ and *x*_*b*′_ from a moving phase *I*_*P*_ to the reference grid (energy grid) *I*_*R*_.

The EMT algorithm used in our online dose reconstruction framework is an adaptation of the method described by Li *et al*.^9^. The working principle of the energy mapping procedure is illustrated in Fig. 3. It shows the energy transfer of two locations *x*′_*a*_ and *x*′_*b*_ from the moving phase *I*_*P*_ to the reference phase *I*_*R*_. The geometrical deformation is characterised by two displacement vectors which map the centre of the source image voxels to the target points *x*_*a*_ and *x*_*b*_ (marked by a green and red cross) in the reference energy grid. Importantly, the target point of a displacement vector does not necessarily have to coincide with the centre of a reference grid voxel. It describes a continuous transformation which is independent of the target grid.

The offset of the target point relative to the nearest reference voxel centre is used to distribute the transformed energy continuously to the reference grid. Figure 3 illustrates how the distribution works on the example of translating source position *x*′_*a*_ to *x*_*a*_ (green cross). The target position of this displacement vector is located in the upper left corner of voxel 5 in the reference grid *I*_*R*_. Scoring all the energy *E*_*T*_ into voxel 5 would result in an improper discretisation of the transformation potentially leading to high gradients and gaps in the accumulated dose. The continuous character of the DVF is conserved by distributing part of the energy also to the neighbouring voxels 1, 2 and 4. The amount of energy the neighbouring voxels receive is determined by a geometrical consideration: An additional virtual voxel (dashed lines) is spanned around the target point *a*_*x*_ which overlaps with the energy voxels of the reference grid. The area of the overlap region (*V*_1−4_) then corresponds to the amount of energy scored to the respective voxels, e.g. the largest fraction of energy is added to voxel 5 while voxel 1 only gets a relatively small share of the transferred energy *E*_*T*_.

Figure 3 shows the principle of our EMT method on a two dimensional example. Extending the principle to 3D is easy due to the highly symmetric character of the geometric consideration. Using this approach on a realistic 3D grid results in 8 overlapping volumes between the three dimensional virtual voxel around the target point and the reference grid. In 3D the number of voxel neighbours doubles since 4 additional overlap regions are formed with the voxels of one of the adjacent slices of the reference grid. The voxels to which the overlap regions belong will be referred to as overlapping voxels hereinafter. The distribution Δ*E*_*j*_ of the transferred energy *E*_*T*_ between the 8 overlap voxels in 3D is calculated by:

1 $${\rm{\Delta }}{E}_{j}=\frac{{V}_{j}}{{V}_{voxel}}\ast {E}_{T},j\in \{\mathrm{1..8}\}$$

with *V*_*j*_ being the overlapping volume between the virtual voxel constructed around the target point and the voxels of the reference grid. *V*_*voxel*_ denotes the total volume of one reference voxel.

The overlap volumes can be easily quantified by the position of the displacement vector relative to the voxel grid which is demonstrated in 2D in Fig. 3. For instance the overlapping area *V*_1_ can be calculated by multiplying the length of Δ*x*_1_ and Δ*x*_2_ which is the offset of the target point from the centre of voxel 5. Since equation 1 only uses the overlapping volumes with respect to the total voxel volume we can (without any loss of generality) make use of relative coordinates:

2 $${\bar{x}}_{i}=\frac{{x}_{i}}{{r}_{i}},i\in \{\mathrm{1..3}\}$$

with *r*_*i*_ being the reference voxel resolution in dimension *i*. Using relative coordinates ${\bar{V}}_{voxel}$ becomes 1 and equation 1 simplifies to:

3 $${\rm{\Delta }}{E}_{i}={\bar{V}}_{j}\ast {E}_{T}$$

According to the example in Fig. 3 the relative overlapping volumes in 2D are calculated to: ${\bar{V}}_{1}={\rm{\Delta }}{\bar{x}}_{1}{\rm{\Delta }}{\bar{x}}_{2}$, ${\bar{V}}_{2}\mathrm{=(1}-{\rm{\Delta }}{\bar{x}}_{1}){\rm{\Delta }}{\bar{x}}_{2}$, ${\bar{V}}_{3}={\rm{\Delta }}{\bar{x}}_{1}\mathrm{(1}-{\rm{\Delta }}{\bar{x}}_{2})$ and ${\bar{V}}_{4}\mathrm{=(1}-{\rm{\Delta }}{\bar{x}}_{1}\mathrm{)(1}-{\rm{\Delta }}{\bar{x}}_{2})$. The example reveals a symmetric pattern which determines the relative overlapping area by multiplying a combination of $({\rm{\Delta }}{\bar{x}}_{1}\mathrm{,1}-{\rm{\Delta }}{\bar{x}}_{1})$ and $({\rm{\Delta }}{\bar{x}}_{2}\mathrm{,1}-{\rm{\Delta }}{\bar{x}}_{2})$. The combination depends on the target position relative to the voxel centre, more precisely on the quadrant of the nearest reference voxel in which the target point is located in the grid. For the example the overlapping areas around point *x*_*b*_ (which is located in the upper right quadrant of voxel 8) are calculated by a different combination of Δ*x*_1_ and Δ*x*_2_. For realistic cases in 3D an additional factor ${\rm{\Delta }}{\bar{x}}_{3}$ has to be considered which describes the relative offset of the target point to the nearest voxel centre in z-direction. With three factors $\Delta {\bar{x}}_{i}$ used in two configurations $({\rm{\Delta }}{\bar{x}}_{i}\mathrm{,1}-{\rm{\Delta }}{\bar{x}}_{i})$ all 2^3^ = 8 overlap voxels can be quantified.

**Figure 4:**
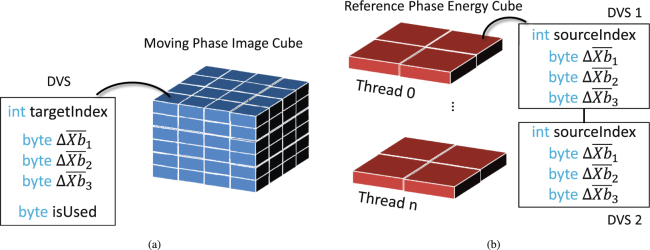
Displacement Vector Structure (DVS) and their arrangement. (**a**) Arranged with the moving image cube for push implementation. (**b**) Arranged with the reference energy cube for pull implementation.

The method for distributing energy to neighbouring voxel developed in this section has two main advantages from a computational point of view. First, only 3 relative coordinates ${\rm{\Delta }}{\bar{x}}_{i}$ have to be stored per displacement vector in order to determine the energy distribution to all 8 neighbouring voxels. Second, the energy distribution *E*_*j*_ is calculated by simple arithmetic operations (multiply and add) which can be executed relatively quickly on modern processors. The data structure in our implementation which describes the energy transfer along one displacement vector is listed on the left hand side in Fig. 4. It will be referred to as displacement vector structure (DVS) hereinafter.

The structure consists of three types of data. First, an index *targetIndex* which specifies the target energy voxel in the reference grid. Second, the three dimensional offset ${\rm{\Delta }}\bar{X}{b}_{i}$ of the displacement vector target relative to the centre of the voxel specified by *targetIndex* and third a status byte *is Used* which indicates whether or not the element should be mapped to the reference grid. The status byte is beneficial if only selected image voxels of a certain region of interest should be transformed (e.g. only voxels inside the patient contour). ${\rm{\Delta }}\bar{X}{b}_{i}$ stores the relative target coordinates as a signed 8-bit integer variable to minimise the memory footprint of the DVS structure. The floating point representation of the coordinates can be retrieved by: ${\rm{\Delta }}{\bar{x}}_{i}={\rm{\Delta }}\bar{X}{b}_{i}{\mathrm{/(2}}^{7}-\mathrm{1)}$. The sign of ${\rm{\Delta }}{\bar{x}}_{i}$ reveals the energy voxel quadrant in which the displacement vector falls and thus defines how to combine the factors $({\rm{\Delta }}{\bar{x}}_{i}\mathrm{,1}-{\rm{\Delta }}{\bar{x}}_{i})$ in order to calculate the overlapping volume ${\bar{V}}_{j}$. The pseudo-code for calculating ${\bar{V}}_{j}$ is provided in the supplementary material Appendix Algorithm 1. Following the ideas developed in this section results in a highly efficient single-threaded energy/mass transfer implementation on modern processors.

### Parallelisation

In the previous section we introduced the basic idea of our performance optimised EMT implementation in a non-parallel processing environment. Even more performance can be achieved by processing the DVF in concurrent tasks using state-of-the-art parallel processors. Parallelising the EMT seems straight forward, but unfortunately the push direction (transporting energy from the moving phase grid to the reference grid) as proposed by Li *et al*.^9^ turns out to be unfavourable for an efficient parallel implementations. Problems arise if tissue from multiple source image voxels are compressed to one voxel in the reference grid (see Figure 1). A so called race condition may occur in this situation if the two energy contributions from the moving phase are scored to the same voxel simultaneously. In this scenario two processor threads would try to add their transferred energy to the reference voxel at the same time which results in the loss of at least one of the contributions. Such a scenario is called a race condition. With the energy distribution scheme introduced in the last section a race condition may not only arise when tissue is compressed but also when the sets of overlapping voxels from two threads are not disjoint like shown in Fig. 3. If both locations at *x*′_*a*_ and *x*′_*b*_ are transferred in parallel the energy contribution to voxel 5 in the reference grid is likely to be corrupt since the same data location must not be altered by two threads at the same time.

An efficient way to prevent race conditions is to avoid concurrent energy scoring to the same location in the first place. This is achieved by reversing the EMT algorithm from a push to a pull process. Instead of looping through the moving phase voxels and pushing their energy contribution to the reference grid, we now loop over the voxels of the reference phase and pull the energy contribution of the moving phase back to the energy grid. The parallelisation decomposes the reference grid into individual fractions of energy voxels which are processed concurrently. This ensures that the energy to one specific reference grid voxel is always updated by the same thread to avoid collisions between them. The DVS data structure needs three alterations to enable a pull approach: First, the target index *targetIndex* needs to be replaced by a *sourceIndex* which points to the image voxel in the moving phase. Second, the DVS elements each describing the transformation along one displacement vector are now arranged with the reference energy grid. One energy voxel gets one or multiple DVS assigned to it which are all processed by the same thread. The right side of Fig. 4 illustrates the new parallel DVS data structure. It shows two DVS elements which are assigned to one energy voxel representing two locations from the moving phase mapped to the same reference voxel. Third, the status word *is Used* is no longer needed since regions of interest can be directly defined in the reference grid. If an energy voxel is processed all its assigned DVS elements should be processed, too.

A data construct as shown on the right side of Fig. 4 can easily be parallelised without encountering any race conditions. In our implementation we decomposed the reference grid along the z-direction and distributed the workload to different threads. Please note that the DVF data did not change. The pull implementation describes the same transformation as the serial push implementation introduced in the previous section. The result of the dose accumulation is the same but the order in which the displacement vector fields are processed was changed in favour of the parallelisation. The algorithm outlined in the supplementary material Appendix Algorithm 1 can be easily adapted for the pull implementation: The *is Used* check can be omitted while the *index* in line 4 is now retrieved simply from the index of the energy voxel to which the DVS is assigned to.

### Vectorisation

In the previous section we discussed thread-level parallelisation of our EMT method which makes use of multiple physical CPU cores. In this section we are going to extend our implementation even further to exploit data-level parallelism (DLP). DLP makes use of wide arithmetic registers within a CPU core. Modern processor cores can perform arithmetic operations on up to 8 values simultaneously. This level of parallelism is usually not implemented manually by the programmer but installed by the compiler. However the programmer needs to structure the algorithm in a useful way so that the compiler can exploit DLP.

**Figure 5:**
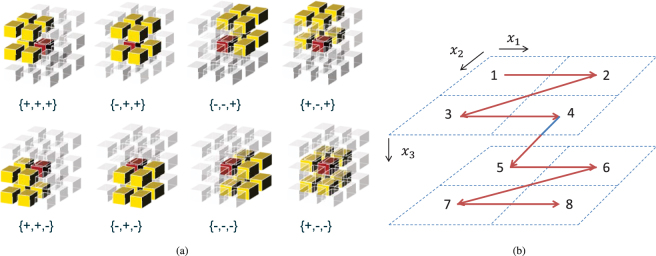
Dose warping schemata. (**a**) All 8 cases of how dose can be warped to the referenced grid relative to the target voxel (red) of a deformable vector element. The cases are labelled by the signs $$\{sign({\rm{\Delta }}\bar{X}{b}_{1}),sign({\rm{\Delta }}\bar{X}{b}_{2}),$$$$sign({\rm{\Delta }}\bar{X}{b}_{3})\}$$ of the corresponding relative target coordinate variables (cf. Algorithm 1 listed in the supplementary material). (**b**) Unified pattern for traversing the overlapping voxel ensemble starting at the smallest voxel index.

The main computational intensive operation in our EMT method is the calculation of the energy distribution *E*_*j*_ according to equation 3. This includes the determination of the energy for every overlapping voxel which are constructed from combining the relative target point coordinates ${\rm{\Delta }}{\bar{x}}_{i}$. The combinatorial scheme depends on the sign of ${\rm{\Delta }}{\bar{x}}_{i}$ for each transformation which is realised by conditional statements (see pseudo code in appendix Algorithm 1 line 9–14). A graphical interpretation of the different schemata is shown in Fig. 5. Every voxel has 26 direct neighbours in three dimensions. Depending on the endpoint of the transformation vector in the target voxel (shown in red), there are 8 different cases or patterns of the overlap region between the transformed voxel and the reference voxel grid.

DLP does not work efficiently on conditional branching which is for instance implemented by using *if… then* statements. The pattern of calculating *E*_*j*_ for the 8 neighbouring voxels from ${\rm{\Delta }}{\bar{x}}_{i}$ needs to be unified for all DVS elements. This is achieved through reducing the combinatorial possibilities to only one specific case by re-arranging the energy distribution scheme as follows: First, the reference point of the energy distribution is changed. Instead of storing the target index of the energy voxel which is closest to the target point, we are now interested in the smallest index among the 8 overlapping voxel ensemble. From that voxel the overlap region is constructed by a fixed scheme stepping at most one voxel successively in the positive direction of each dimension. The fixed scheme is shown in Fig. 5. It maps all 8 cases to one unified pattern to create the overlapping area.

The voxels are traversed and numbered according to their position index (from smallest to largest) in the energy grid, starting from the reference voxel 1 which has the smallest index and ending at voxel 8 which is located at an offset of one voxel in each dimension relative to voxel 1. Second, the relative target positions ${\rm{\Delta }}{\bar{x}}_{i}$ are replaced by three weighting factors ${w}_{i}^{0}$ (*i*∈1..3). The values for ${w}_{i}^{0}$ are calculated from ${\rm{\Delta }}{\bar{x}}_{i}$ in a way so that the overlapping volume of the reference voxel 1 always amounts to ${\bar{V}}_{1}={w}_{1}^{0}\ast {w}_{2}^{0}\ast {w}_{3}^{0}$. A step towards the next voxel in dimension *i* changes the corresponding weighting factor to ${w}_{i}^{+}\mathrm{=1}-{w}_{i}^{0}$ so that for instance the overlapping volume of voxel 2 results in ${\bar{V}}_{2}={w}_{1}^{+}\ast {w}_{2}^{0}\ast {w}_{3}^{0}$, etc. This new calculation scheme for ${\bar{V}}_{i}$ does not use any conditional statement and can be easily implemented in both mapping methods, the single-threaded push method and the parallelised pull method: The DVS used in the push method already provides an indexing variable which is simply overwritten with the index of the reference voxel 1. When using the pull method, the assignment of the DVS elements to the energy voxels has to be changed. Instead of storing DVS elements at the position of the target voxel of the displacement vector, we now assign DVS elements to the energy voxel which has the same index as the reference voxel.

All 8 energy distribution to the overlapping voxels can be calculated simultaneously using the advanced vector extension (AVX) capability of modern CPUs. AVX provides arithmetic operations on 256 bit wide registers that can be populated with 8 single precision float point values (32 bit each). The pseudo-code for a vectorised energy mapping for one DVS element using AVX is given in in the supplementary material Algorithm 2. The mapping function is designed for the pull algorithm using the DVS shown on the right side of Fig. 4 but with weighting factors ${w}_{i}^{0}$ instead of ${\rm{\Delta }}{\bar{x}}_{i}$. The function takes three input parameters, the dose at the location in the moving phase which should be mapped, the index of the reference overlapping voxel and the weighting factors ${w}_{i}^{0}$ which were introduced in this section. These three parameters basically represent the content of one DVS element. The algorithm starts in line 2 by constructing a vector Δ*I*^*AVX*^ that holds the indices of all 8 overlapping voxels relative to the reference voxel. The entries are sorted, starting from the reference voxel 1 and following the pattern shown in Fig. 5. The absolute indices *I*^*AVX*^ of the overlapping voxel set are calculated in line 6 by adding the index of the voxel 1. The function *Vec*(*index*) creates a vector (8 entries) in which every element is set to the value of the scalar parameter *index*. The operator ⊕ performs an element-wise add between two vectors. The energy distribution *E*^*AVX*^ of the overlap area is calculated in line 7 with the help of vectors ${X}_{i}^{AVX}$ describing the overlapping volumes by combining the distribution factors ${w}_{i}^{0}$. The energy contributions are then derived by an element-wise multiplication ($\odot$) of the vectors ${X}_{i}^{AVX}$ and the vectorised energy contribution (*Vec*(*d* * *ed* * *V*_*A*_)) from the source image voxel. *I*^*AVX*^ and *E*^*AVX*^ are now storing the absolute indices and energy contributions to all 8 overlap voxels described by one displacement vector field element. The contributions are scored into the reference energy grid in line 8–10 by looping over the vector elements.

By eliminating conditional branching from the energy distribution calculation using vectorised operations was possible. The vectors created in the pseudo-code are mapped directly to the AVX registers in the CPU by using compiler intrinsics. The vectorised operations $\odot$ and ⊕ are implemented in hardware on the AVX instruction set and can be executed within only a few CPU clock cycles.

### Patient data, treatment delivery and computational platform

**Figure 6:**
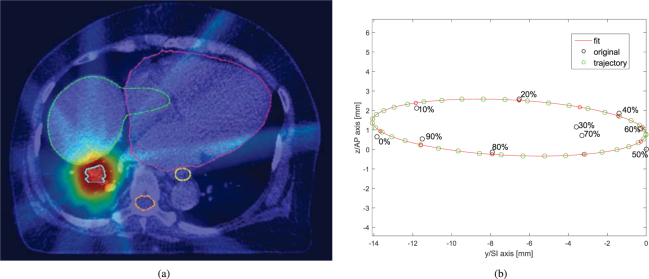
Lung patient case used to assess the performance of our EMT implementations. (**a**) A transversal slice of CT is shown with a dose wash overlay characterising the accumulated dose. The contours represent local anatomy: eusophagus (yellow), spinal cord (orange), liver (green) and heart (red). (**b**) motion trajectory of the tumour target.

The performance of all three (serial, parallel and parallel + vectorised) implementations of our EMT algorithm was tested for a typical 4D lung data set. The treatment was delivered on an Elekta Synergy research linac equipped with an Agility MLC using the dynamic MLC tumour tracking delivery technique^13^. The target motion was simulated during delivery by approximating the 10 centre-of-target positions from the 4D CT with a continuous ellipse. The resulting accumulated dose and the trajectory of the target are shown in Fig. 6.

The periodic movement due to the breathing cycle of the patient is divided into 10 phases following the convention of the phased-binned 4D planning CT. The phase images and the displacement vector fields are stored at a resolution of 1 × 1 × 2 *mm*^3^ amounting to 512 × 512 × 173 voxels while the phase dose, the deformed mass and the reference energy grid are calculated on a 2 × 2 × 2 *mm*^3^ cube which consists of 256 × 256 × 173 voxels. The transformation of each moving phase to the reference grid is described by 11.2 million displacement vectors. The dose accumulation was performed on the whole patient volume between the first and last slice containing lung tissue. Each 4DCT phase was registered to the reference phase (peak exhale) using RayStation’s hybrid DIR module utilising image intensities, lung delineations, and patient contour^14,15^.

Runtimes were acquired on a dual Xeon E5-2697v3 workstation with 64 GB of RAM. The EMT implementations were embedded into our in-house dose reconstruction framework which runs on Windows 7 OS and was compiled in C++ on Visual Studio 2010 professional. The vectorised version of our energy mapping was implemented using Microsoft Compiler Intrinsics.

### Data availability

The patient data that support the findings of this study was acquired during routine clinical practice at The Royal Marsden NHS Foundation Trust. The patients consented for their images to be used for research at The Institute of Cancer Research and publication of these results.

## Results

**Figure 7:**
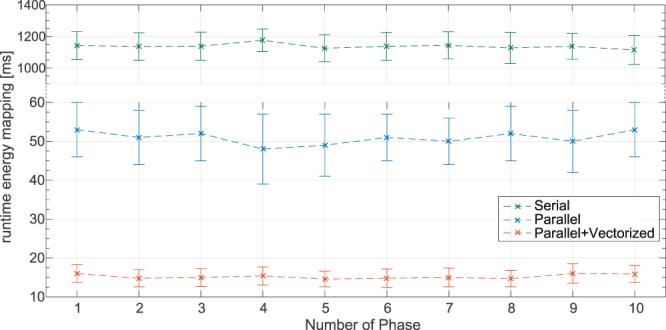
Mean runtimes of the energy mass transfer implementations for all phases of the breathing cycle. The standard deviation was calculated from 1000 repetitions of each measured data point.

The measured runtimes of our intuitive serial EMT implementation, the parallel multi-core EMT implementation and the fully parallel and vectorised version introduced in the last subsection of the materials are shown in Fig. 7 for all respiratory phases of the patient motion.

Table 1 lists the runtimes displayed in Fig. 7 and corresponding memory bandwidth for three phases. The last column of the table states the time which is needed to divide the reference energy cube by the transferred mass in order to yield accumulated dose. This process which corresponds to (4) in Fig. 2 was not discussed in depth in this paper as it is relatively simple to implement.

**Table 1:** Runtime T and bandwidth BW of the serial, parallel and vectorised EMT implementations for phases 1,5 and 10. The last column of the table reports on the time it takes to divide the reference energy grid by the transferred mass distribution yielding the accumulated dose.

#Phase	Serial		Parallel		Parallel + Vectorised		E/m
	T [ms]	BW [GB/s]	T [ms]	BW [GB/s]	T [ms]	BW [GB/s]	T [ms]
1	1142 ± 89	0.75	53 ± 7	17.8	16.0 ± 2.3	58.9	3.5
5	1125 ± 85	0.76	49 ± 8	19.2	14.6 ± 2.0	64.6	3.4
10	1114 ± 91	0.77	53 ± 7	17.8	15.9 ± 2.2	59.3	3.4

It has been verified that all three implementations of the EMT algorithm yield the same dosimetric result. The mean deviation of the parallel implementation relative to the serial implementation was 4.1 × 10^−5^% while the vectorisation added a mean error of 4.5 × 10^−5^% to the dose of each voxel in the reference grid.

## Discussion

In this study we present a real-time CPU-based energy/mass transfer mapping method for online 4D dose reconstruction. The EMT method is implemented in three variations which exploit different levels of parallelism provided by the CPU hardware. The serial implementation does not explicitly use any parallelisation, however the described algorithm is tailored to run efficiently on modern CPUs. It can be implemented relatively easy without any specific knowledge about parallel hardware. The parallel implementation is more advanced and involves significant adoptions of the data structures and a turn-around of the transfer direction from push to pull. This study demonstrates that highly parallel and high performance oriented algorithms are not generated automatically by the compiler alone. Instead the working principles of the underlying hardware and parallel mechanisms need to be exploited to enforce optimal performance. The fastest implementation makes use of parallelisation and vectorisation and enables true real-time dose reconstructions. The key features of our EMT implementation are explained in detail so that they can be re-implemented easily.

The runtimes shown in Fig. 7 and listed exemplary in Table 1 demonstrate the importance of using parallelism in modern processors. The parallel implementation realises a speed-up of about 22x compared to the serial implementation which is reasonable on a 28 core workstation. A perfect linear scaling (i.e. 28x) cannot be expected due to multiple reasons. First, the CPU reduces its clock frequency to control power consumption and heat emission when several or all cores are used. Second, the EMT algorithm is expected to turn into a memory-bound problem after parallelisation which does not scale any further even if more computational cores are employed. The vectorisation of the EMT method adds another 3.3x in performance which is a vital improvement in order to use the EMT within our dose reconstruction workflow that updates actually delivered dose at 25 Hz. The total speed-up of about 72x which was achieved by combining multi-threading and vectorisation demonstrates the performance potential of using parallelism in modern CPUs.

The fastest implementation transfers displacement vector and dose/energy data with up to 64 GB/s from memory to CPU on a system which can practically reach about 80 GB/s. One can conclude that the runtime of the algorithm is dominated by transporting data between main memory and CPU cores while the time spend on arithmetic calculations is relatively small. This is due to the following reason: First, The EMT algorithm performs only a few relatively simple arithmetic operations on a large set of data. For example the vectorised implementation shown in algorithm 2 executes four arithmetic vector operations (line 6, 7) on 21 data words which need to be fetched and written to memory. This imbalance reveals its negative effect on the performance since simple arithmetic operations especially on vectorised data are usually much faster than fetching data from memory. The EMT algorithm is said to be memory-bound and it is important that the memory footprint of the pre-calculated data is kept to a minimum. The data structures introduced in Fig. 4 are carefully designed to meet exactly this requirement. Simpler data-structures are likely to produce significantly more data overhead which translates to expected longer runtimes. Intuitively, one could suggest for instance to store the displacement vector field in form of an uncoupled linear structure similar to a dose influence matrix. The elements of such a matrix would contain a weight and two indices denoting the target and source voxel, respectively. Processing this matrix in parallel would be possible since elements could be easily sorted so that they do not overlap. However, the memory footprint would be tremendous. Each matrix element would need to store 2 bytes for the weight, 4 bytes for the target voxel index and 4 byte for the reference voxel index which makes 80 byte per displacement vector field (10 byte element for 8 target voxel) compared to 7 to 8 byte used in our method as shown in Fig. 4. In this work we implemented a vectorised algorithm for the AVX instruction set. An AVX register can hold and process 8 single precision float vales at once which coincides with the number of overlapping dose regions of the energy voxel. However, the presented EMT algorithm does not require AVX capabilities nor is it limited to 8 overlapping voxel regions either. More than 8 elements can be processed in subsequent operations through using two or more AVX registers. In the same way, SSE instruction sets which only offer a vectorisation of 4 data elements can be used: One AVX operation can easily be split in two SSE operations with a possible performance loss.

The plots in Fig. 7 show that the runtime of the EMT is the same for all respiratory phases (within errors). In other words, the actual form of the displacement vector field and the dose distribution have no influence on the runtime which is a pre-requisite to use the algorithm in a real-time workflow that allocates a fixed time slot. The difference between all three EMT implementations is in the order of 10^−5^%. This indicates that the parallel and vectorised EMT implementation perform the same operations. The small deviations are due to numerical uncertainties since the order of the energy mapping is different. The general accuracy of EMT (and DDM & any other dose warping method) is limited by the DIR performance. Therefore it is imperative that the DIR is properly validated^16^.

The mapping method used for our implementation does only rely on translational shifts of single image voxels in order to transfer energy and mass between the phases. Rotations and deformations of voxels were not considered. Handling irregularly shaped voxels is more complex and thus requires more computational effort. While our model consists of simple steps that can be parallelised and vectorised efficiently, irregular voxel boundaries would need additional memory and geometric calculations to identify the overlap with the reference grid. This is currently not feasible on affordable computational hardware while satisfying the same real-time constraint (25 Hz) we set for this study. We would also argue that local deformations and rotations of the patient geometry can be closely approximated by a series of image voxel translations. This holds especially true if the voxels are small enough, for instance 1 mm × 1 mm × 2 mm as used in this work. A similar argumentation can be found in Li *et al*.^9^.

The objective of this work was to design a real-time EMT mapping implementation which integrates seamlessly into an online dose reconstruction workflow as shown in Fig. 2. This goal is best achieved on a CPU. Using accelerators such as GPUs for the computation of EMT would not result in such a high overall performance as the GPU has its own physical memory which makes additional data transfers necessary. In case of a GPU-based EMT implementation embedded in our dose reconstruction workflow this would mean that at least the phase dose cube and DVS data has to be loaded onto the GPU before the dose mapping can start and the updated energy grid needs to be transferred back to the CPU memory afterwards. Even on modern GPUs this would add an additional transfer overhead which is in the order of the actual EMT runtime. This bottleneck around the data transfer would nullify the real-time character of the EMT algorithm.

It has been shown that offline ART considerably improves the accuracy of RT of lung cancer^17^. Previous studies^18^ demonstrated the benefit of online plan adaptation by monitoring anatomical changes in 233 lung patients online during the treatment using cone-beam CT-scans. The treatment was interrupted in order to create a new plan if systematic changes above a pre-defined trigger criteria were observed. During the study it was found that 27% of the patients needed a plan adaptation which lead to a significant decrease in lung dose (mean lung dose reduced from 14.6 Gy to 12.6 Gy on average). It is reported that 75% of the plan adaptations corrected a decrease in target coverage or an overdosage of the spinal cord.

The next step for online ART is adaptation during delivery, which is becoming an area of research with the advent of new treatment concepts (e.g. MR linac) allowing for real-time anatomy detection. For example a 4D dose reconstruction workflow has been proposed^19^ based on pseudo 4DCT generated from online MR imaging and found local underdosage of 2 Gy and overdosages up to 1.5 Gy in the PTV. Local variations in the nearby organs-at-risk (OAR), which are discussed as significant, are reported to range from −1.3 to 1.9 Gy. The dose reconstruction algorithm used in their work utilises a Monte Carlo simulation which takes up to 15 seconds per MLC aperture and hence cannot be applied to an online dose reconstruction scenario. The authors do not report the runtime of their dose accumulation method. Our implementation of EMT could be utilised in their setting. In our work on real-time 4D dose reconstruction for lung SBRT^11^ we could show that dose reconstruction at 25 Hz can be realised on a single workstation. Decreased PTV margins resulted in inadequate target coverage during untracked deliveries for patients with substantial tumour motion. MLC tracking could ensure the GTV target dose for these patients. OAR doses were consistently reduced by decreased PTV margins. The respective framework included the EMT algorithm as presented here.

## Conclusions

We have implemented a speed-optimised version of the energy/mass transfer mapping method using modern multi-core CPUs. Our fully parallelised and vectorised implementation achieves a speed-up of up to 72x compared to a smart serial implementation of the same algorithm. This demonstrates that optimised code for parallel processor architectures holds a significant performance potential which is often left unexploited. Especially for low latency and real-time applications using CPUs can have many advantages compared to GPUs. The performance of the EMT algorithm as presented in this work is memory-bound. Under that assumption we conclude that there is no significantly faster implementation on commodity shared memory systems since the maximum available memory bandwidth is almost reach. It could be shown that it is possible to reconstruct dose for a lung SBRT patient in real-time using a 4D CT data set on full resolution. A real-time dose reconstruction method as described in this work opens the door to online tracking and evaluation of the actual delivered dose during the treatment as well as online re-planning scenarios.

## Electronic supplementary material

Pseudo Code of EMT Algorithms
