# Supplementary material for: Real-time energy/mass transfer mapping for online 4D dose reconstruction
Source: Sci Rep. 2018 Feb 26;8:3662. doi: 10.1038/s41598-018-21966-x (PMC5827544; doi:10.1038/s41598-018-21966-x)
Supplement: Supplementary file 1 — Pseudo Code of EMT Algorithms [file 41598_2018_21966_MOESM1_ESM.pdf]

Real-time energy/mass transfer mapping for online 4D dose reconstruction

Peter Ziegenhein[1,*], Cornelis Ph. Kamerling[1], Martin F. Fast[1,], and Uwe Oelfke[1]

[1]Joint Department of Physics at The Institute of Cancer Research and The Royal Marsden NHS Foundation Trust, London, UK SM2 5NG
[*]peter.ziegenhein@icr.ac.uk

ABSTRACT

In this work we describe an ultra-fast, low-latency implementation of the energy/mass transfer (EMT) mapping method to accumulate dose on deforming geometries such as lung using the central processing unit (CPU). It enables the computation of the actually delivered dose for intensity-modulated radiation therapy on 4D image data in real-time at 25 Hz. In order to accumulate the delivered dose onto a reference phase a pre-calculated deformable vector field is used. The aim of this study is to present an online dose accumulation technique that can be carried out in less than 40 ms to accommodate the machine log update rate of our research linac. Three speed optimization strategies for the CPU are discussed: single-core optimization, parallelisation for multiple cores and vectorization. The single-core implementation accumulates dose in about 1.1 s on a typical high resolution grid for a lung stereotactic body radiation therapy case. Adding parallelisation decreased the runtime to about 50 ms while adding vectorization satisfied our real-time constraint by further reducing the dose accumulation time to 15 ms without compromising on resolution or accuracy. The presented method allows real-time dose accumulation on deforming patient geometries and has the potential to enable online dose evaluation and re-planning scenarios.

Appendix: Pseudo-code of the energy/mass transfer mapping methods presented

Algorithm 1 Energy mapping and distribution

function MAPPING(DVS)

input: DVS, displacement vector structure describing the transformation of one image location
output: *energyCube*, scoring energy to the overlapping voxel in the reference energy cube

local variables: d, moving phase dose at location x' where the displacement vectors starts
ed, density of moving phase image location x' where displacement vector starts
$index$, index of the target energy voxel, equals DVS.targetIndex
$\Delta\bar{X}b_i$, relative target coordinates, equals DVS.$\Delta\bar{X}b_i$
ix, iy, iz, voxel coordinates of the target energy voxel
x, y, z, overlapping voxel coordinates
$w_{x,y,z}$, energy distribution weighting factor for overlapping voxel
$dimX, dimY$, dimensions of the reference energy cube
j, overlapping voxel index
E_j, energy fraction written to overlapping voxel j

  if (DVS.isUsed is **false**) **then** exit
  end if
  split(index,ix,iy,iz) ▷ split target voxel index into voxel coordinates
  $d \leftarrow$ doseAt(DVS)
  for $dz = 0..1$ **do** ▷ looping overlap voxels
    for $dy = 0..1$ **do**
      for $dz = 0..1$ **do**
        $z \leftarrow \Delta\bar{X}b_3 > 0 \ ? \ (iz + dz) : (iz - dz)$ ▷ if $\Delta\bar{X}b_3 > 0$ then $z = iz + dz$
        $y \leftarrow \Delta\bar{X}b_2 > 0 \ ? \ (iy + dy) : (iy - dy)$ ▷ if $\Delta\bar{X}b_2 <= 0$ then $y = iy - dy$
        $x \leftarrow \Delta\bar{X}b_1 > 0 \ ? \ (ix + dx) : (ix - dx)$

        $w_z \leftarrow (dz == 0) \ ? \ (127 - \Delta\bar{X}b_3)/127 : \Delta\bar{X}b_3/127$
        $w_y \leftarrow (dy == 0) \ ? \ (127 - \Delta\bar{X}b_2)/127 : \Delta\bar{X}b_2/127$
        $w_x \leftarrow (dx == 0) \ ? \ (127 - \Delta\bar{X}b_1)/127 : \Delta\bar{X}b_1/127$

        $j \leftarrow z * dimX * dimY + y * dimX + x$ ▷ calculates voxel index of (x,y,z)
        $E_j \leftarrow d * w_x * w_y * w_z * ed$
        $inc(energyCube[j], E_j)$
      end for
    end for
  end for
end function

Algorithm 2 Vectorised AVX calculation of the energy distribution

function VECTORISEDMAPPING(d,$index$,w_i^0)

input: d, moving phase dose at location x' where the displacement vectors starts
w_i^0, energy distribution weighting factors $i \in [1..3]$
output: $energyCube$, scoring energy to the overlapping voxel in the reference energy cube

local variables: ed, density of moving phase image location x' where displacement vector starts
V_a, volume of one source image voxel
n_x, n_y, number of voxels in X and Y dimension of the reference energy grid
$n_{xy} = n_x * n_y$
$w_i^+ = 1 - w_i^0$

$\Delta I^{AVX} = (0, 1, n_x, n_x + 1, n_{xy}, n_{xy} + 1, n_{xy} + n_x, n_{xy} + n_x + 1)$

$X_1^{AVX} \leftarrow \left(w_1^0, w_1^+, w_1^0, w_1^+, w_1^0, w_1^+, w_1^0\, w_1^+\right)$
$X_2^{AVX} \leftarrow \left(w_2^0, w_2^0, w_2^+, w_2^+, w_2^0, w_2^0, w_2^+\, w_2^+\right)$
$X_3^{AVX} \leftarrow \left(w_3^0, w_3^0, w_3^0, w_3^0, w_3^+, w_3^+, w_3^+\, w_3^+\right)$

$I^{AVX} \leftarrow Vec(index) \oplus \Delta I^{AVX}$
$E^{AXV} \leftarrow Vec(d * ed * V_a) \odot (X_1^{AVX} \odot X_2^{AVX} \odot X_3^{AVX})$

for $j = 1..8$ **do** ▷ looping overlap voxels
$inc\left(energyCube[I^{AVX}[j]], E^{AVX}[j]\right)$ ▷ score energy distribution to reference phase
end for
end function
